# Reward processing in autism spectrum disorder and psychopathy: a systematic review

**DOI:** 10.1192/bjo.2021.160

**Published:** 2021-06-18

**Authors:** Patrick McLaughlin, Marija-Magdalena Petrinovic, Nigel Blackwood

**Affiliations:** IoPNN King's College London

## Abstract

**Aims:**

Emerging research suggests that aberrant reward processing may underpin much of the social dysfunction we see in psychiatric disorders. Two conditions associated with marked social dysfunction are Autism Spectrum Disorder (ASD) and Psychopathy. However, no review to date has directly contrasted reward processing in both conditions and incorporated literature on social and non-social rewards. This systematic review aims to: (i) identify and compare reward processing abnormalities in ASD and Psychopathy as demonstrated in task-based functional magnetic resonance imaging (fMRI) studies; and (ii) identify correlations between fMRI reward processing abnormalities and manifested symptoms, with a focus on those giving rise to social dysfunction.

**Method:**

The electronic databases PubMed, PsycINFO and EMBASE were searched to identify studies satisfying the following criteria: (i) a validated measure was used to assess ASD or Psychopathy; (ii) the study was published in an English language peer review journal; (iii) the age of participants was 18 years or older; (iv) individuals participated in a reward-based experimental paradigm; and (v) the response to the reward was measure using fMRI.

**Result:**

A total of 12 articles were identified that satisfied inclusion criteria. Six studies examined reward processing in ASD and six studies examined reward processing in Psychopathy. All studies in both conditions indicated some degree of abnormal reward-related neural response. The most replicated findings were aberrant responses in the Ventral Striatum (VS). Autism Spectrum Disorder was typified by VS hypoactivation to social and non-social reward, while Psychopathy was associated with VS hyperactivation in response to non-social reward anticipation. No studies were identified of social reward in Psychopathy.

**Conclusion:**

The reported fMRI findings correlate with clinical observations in both conditions. Reduced reward response in ASD to a range of social and non-social stimuli would provide a parsimonious account of the social and non-social deficits that characterise the condition. Enhanced responses to the anticipation of reward in Psychopathy provides an account of the ruthless and destructive pursuit of reward-driven behaviours not inhibited by immoral or aversive signals. If, as the literature suggests, reward circuitry dysfunction plays a role in the development and manifestation of symptoms in both conditions, reward processing and its underlying neural circuitry may represent important targets for the development of novel treatment strategies.

